# Endoscopic ultrasound-guided choledochoduodenostomy results in fewer complications than percutaneous drainage following failed ERCP in malignant distal biliary obstruction

**DOI:** 10.1055/a-2580-1316

**Published:** 2025-05-21

**Authors:** Mike J.P. de Jong, Foke van Delft, Erwin-Jan M. van Geenen, Auke Bogte, Robert C. Verdonk, Niels G. Venneman, Jan Maarten Vrolijk, Jan-Willem A. Straathof, Rogier P. Voermans, Rina A. Bijlsma, Sjoerd D. Kuiken, Rutger Quispel, Muhammed Hadithi, Kirill Basiliya, Frank P. Vleggaar, Tanya M. Bisseling, Thomas R. de Wijkerslooth, Marco J. Bruno, Roy L.J. van Wanrooij, Peter D. Siersema

**Affiliations:** 1 6034 Gastroenterology and Hepatology, Radboud University Medical Center, Nijmegen, Netherlands; 2 6028 Research and Development, St Antonius Hospital, Nieuwegein, Netherlands; 3 8124 Gastroenterology and Hepatology, UMC Utrecht, Utrecht, Netherlands; 4 6028 Gastroenterology and Hepatology, St Antonius Hospital, Nieuwegein, Netherlands; 5 3231 Gastroenterology and Hepatology, Medisch Spectrum Twente, Enschede, Netherlands; 6 1322 Gastroenterology and Hepatology, Rijnstate Hospital, Arnhem, Netherlands; 7 89569 Gastroenterology and Hepatology, Maxima Medical Centre, Veldhoven, Netherlands; 8 522567 Gastroenterology and Hepatology, Amsterdam University Medical Centres, Amsterdam, Netherlands; 9 61363 Gastroenterology and Hepatology, Martini Hospital, Groningen, Netherlands; 10 10215 Gastroenterology and Hepatology, Onze Lieve Vrouwe Gasthuis, Amsterdam, Netherlands; 11 84744 Gastroenterology and Hepatology, Reinier de Graaf Gasthuis, Delft, Netherlands; 12 7000 Gastroenterology and Hepatology, Maasstad Hospital, Rotterdam, Netherlands; 13 4501 Gastroenterology and Hepatology, Leiden University Medical Center, Leiden, Netherlands; 14 8124 Gastroenterology and Hepatology, University Medical Centre Utrecht, Utrecht, Netherlands; 15 1228 Gastrointestinal Oncology, Netherlands Cancer Institute, Amsterdam, Netherlands; 16 6993 Gastroenterology and Hepatology, Erasmus MC University Medical Center Rotterdam, Rotterdam, Netherlands; 17 1209 Gastroenterology and Hepatology, Amsterdam UMC Locatie VUmc, Amsterdam, Netherlands

## Abstract

**Background:**

Percutaneous transhepatic biliary drainage (PTBD) and endoscopic ultrasound-guided biliary drainage (EUS-BD), including choledochoduodenostomy (EUS-CDS), are alternative methods for biliary drainage in patients with distal malignant biliary obstruction (MBO) after failed endoscopic retrograde cholangiopancreatography (ERCP). Data on long-term outcomes, adverse events (AEs), and quality of life (QoL) after EUS-CDS and PTBD are limited. Therefore, we created a registry to evaluate the outcomes of both drainage procedures.

**Methods:**

Patients with distal MBO who underwent EUS-CDS or PTBD after unsuccessful ERCP were included in this multicenter investigator-initiated prospective registry over an 18-month inclusion period. Primary end points were procedure-related AEs and mortality within 90 days post-procedure. Secondary end points included technical and clinical success, reinterventions, hospital stay, and QoL.

**Results:**

55 patients were included, with 12 patients undergoing PTBD (technical success 100%) and 43 patients EUS-CDS (technical success 97.7%). Prior to ERCP, 7/12 patients in the PTBD group and 12/43 patients in the EUS-CDS group opted for best supportive care. The 90-day mortality rate was 66.7% in the PTBD group and 20.9% in the EUS-CDS group ( *P* = 0.005). Furthermore, 11/12 patients (91.7%) in the PTBD group and 19/43 (44.2%) in the EUS-CDS group developed one or more AEs ( *P* = 0.004). The median post-procedural hospital stay was 4 days (interquartile range [IQR] 2–6) in the PTBD group vs. 1 day (IQR 1–2) in the EUS-CDS group ( *P* = 0.001).

**Conclusion:**

When both modalities were available and technically feasible, gastroenterologists preferred EUS-CDS over PTBD. EUS-CDS seems to be associated with lower mortality and AE rates, shorter hospital admission, and fewer reinterventions, but a randomized controlled trial should confirm these observations.

## Abstract (de)

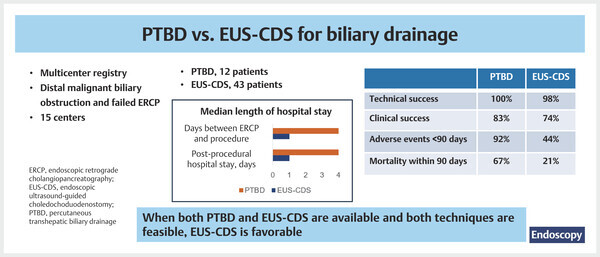

## Introduction

Distal malignant biliary obstruction (MBO) is predominantly caused by pancreatic adenocarcinoma or cholangiocarcinoma 1 . The obstruction of the common bile duct (CBD) leads to jaundice, which is present in approximately 70% of patients diagnosed with pancreatic carcinoma 2 . For all primary tumors causing bile duct obstruction, cure can be achieved only by surgical resection, whether or not it is preceded or followed by systemic treatment 3 . It is essential to ensure adequate biliary drainage for preoperative episodes of cholangitis or when neoadjuvant systemic treatment is administered 4 . Otherwise, many chemotherapeutic agents are contraindicated as reduced hepatic clearance can lead to increased toxicity 5 .

Transpapillary placement of a self-expanding metal stent (SEMS) during endoscopic retrograde cholangiopancreatography (ERCP) is the gold standard to achieve adequate bile duct drainage 6 . Unfortunately, approximately 12.5% of these procedures do not succeed owing to tumor-related anatomical or technical difficulties 7 . Percutaneous transhepatic biliary drainage (PTBD) is the historical alternative method of draining the biliary tree 6 . Its disadvantages are significant 30-day overall mortality and morbidity rates, ranging from 17% to 23% and 40% to 70%, respectively 8 9 10 11 12 .

Endoscopic ultrasound-guided choledochoduodenostomy (EUS-CDS) with placement of a lumen apposing metal stent (LAMS) is an emerging alternative modality for biliary drainage 13 . Compared with the high level of AEs for the PTBD, EUS-CDS has an AE rate of 7%–36% 14 15 16 . Despite its lower AE rate, EUS-CDS is not always superior to PTBD, as EUS-CDS is not always technically feasible. When both drainage modalities are available and technically feasible, the European Society of Gastrointestinal Endoscopy (ESGE) recommends performing an EUS-guided biliary drainage (EUS-BD) procedure instead of PTBD after a failed ERCP 13 . EUS-BD has demonstrated similar technical success, but fewer AEs and higher clinical success 17 ; however, the studies incorporated in the guideline were mainly retrospective single-center studies using different techniques and stents, and were performed in tertiary centers, resulting in a moderate quality of evidence 13 .

Currently, there is still a lack of data on the long-term clinical and technical outcomes of the two alternative biliary drainage techniques, and on quality of life (QoL) after a failed ERCP in patients with distal MBO. The BESTDRAIN registry aims to provide insight into the chosen drainage modality after a failed ERCP procedure and evaluates prospectively the clinical outcomes, QoL, and AEs of both PTBD and EUS-CDS in both tertiary academic and community hospitals in The Netherlands.

## Methods

### Study design

This study was designed as a prospective investigator-initiated multicenter registry. Patients were included in 15 hospitals (four academic and 11 community hospitals) in The Netherlands during a study period of 18 months from July 2022 till the end of December 2023. All participating hospitals were able to perform both EUS-CDS and PTBD.

This study followed the principles of the Declaration of Helsinki 18 . It was approved by the medical ethical committee (#2020–6758) of the Radboud University Medical Center, Nijmegen, The Netherlands and by each local institutional review board. Written informed consent was obtained from all the participating patients.

### Patients

Patients aged 18 years or older, with distal MBO caused by either resectable or unresectable tumors, and failure of biliary drainage during ERCP, were screened for eligibility. Failed biliary drainage was defined as failure to reach the papilla of Vater or to cannulate the CBD, or inability to insert a SEMS after successful cannulation. Patients who had undergone previous PTBD and/or EUS-CDS were excluded, as well as patients who were American Society of Anesthesiologists (ASA) grade IV or V, pregnant women, and patients who were unable to provide informed consent. Patients with ASA IV and V were excluded to minimize the potential selection bias, as we were concerned that ASA IV and V patients would be preferentially referred to interventional radiology for PTBD, which could introduce bias in comparing the two treatment modalities.

### Procedure

**Fig. 1:**
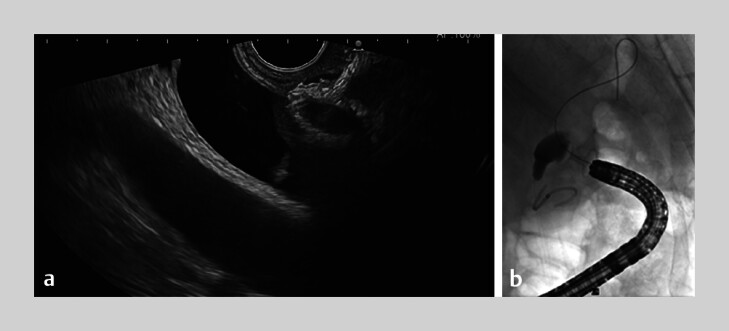
Images during endoscopic ultrasound-guided choledochoduodenostomy (EUS-CDS) showing: **a** on EUS image, a lumen-apposing metal stent (LAMS) being inserted into the common bile duct (CBD); **b** on fluoroscopic image, a LAMS inserted between the duodenum and CBD, with a guidewire inserted through the LAMS toward the hilum of the liver for placement a plastic double-pigtail stent.

After obtaining written informed consent, data regarding patients’ medical history, medication use, and the failed ERCP procedure were collected. It was at the physician’s discretion whether a patient underwent EUS-CDS ( Fig. 1 ) or PTBD. The rationale behind the choice between the two drainage modalities was also registered. Therefore, this registry had no impact on standard patient care. Same-session EUS-CDS was not universally available at the participating centers. Any decision on timing depended on institutional protocols and available expertise.

### Outcomes

The primary outcomes were the AE and mortality rates of both EUS-CDS and PTBD within 90 days of the procedure. Cholangitis was scored according to the Tokyo guidelines 19 and acute pancreatitis according to the revised Atlanta criteria 20 . The severity of AEs was graded according to the AGREE classification 21 .

The secondary outcomes were: technical success, sustained clinical success, the interval between ERCP and the alternative drainage modality, hospital admissions, duration of hospital admission, time to reintervention, number of reinterventions, and QoL. Technical success was defined as the ability to place a stent or drain in the bile duct. Stent or drain patency was scored as the number of stents/drains that remained in place without migration or removal within 30 and 90 days. Sustained clinical success was defined as a >50% reduction in plasma total bilirubin levels at the end of 30 days post-procedure.

### Data collection

Data were collected at 72 hours, 30, 90, and 180 days post-procedure. The following data were collected about the first 72 hours post-procedure: route and technique of biliary drainage, technical success, per-procedural AEs, interval between ERCP and the procedure, and the duration of hospital admission. Data regarding AEs, interventions, hospital admissions, bilirubin reduction, and survival outcomes were collected at 30, 90, and 180 days after the PTBD or EUS-CDS. Furthermore, patients were asked to complete the validated Short Form Health Survey (SF)-36 and EuroQol-5 dimensions (EQ-5D) questionnaires at these timepoints 22 23 .

If a patient underwent pancreaticoduodenectomy or died before the end of follow-up, data were no longer collected. Data collection was terminated after surgery, as no stent or drain remained in place after the procedure, making it impossible to further compare the drainage modalities. All data were collected and stored in the CastorEDC data management system (Castor Electronic Data Capture, Ciwit BV, Amsterdam, The Netherlands).

### Statistical analysis

After the last patient visit, the database was locked and data were exported from CastorEDC to SPSS (IBM SPSS Statistics for Windows, version 29; IBM Corp. Armonk, New York, USA) for statistical analysis.

Continuous variables were summarized with standard descriptive statistics including mean (SD), median and range, depending on the skewness of the data. Categorical variables were summarized with frequencies. Survival was presented in Kaplan–Meier curves. A *P* value <0.05 was considered statistically significant. *P* values were calculated using the Mann–Whitney *U* test, Fisher’s exact test, or chi-squared test when appropriate.

## Results

**Fig. 2:**
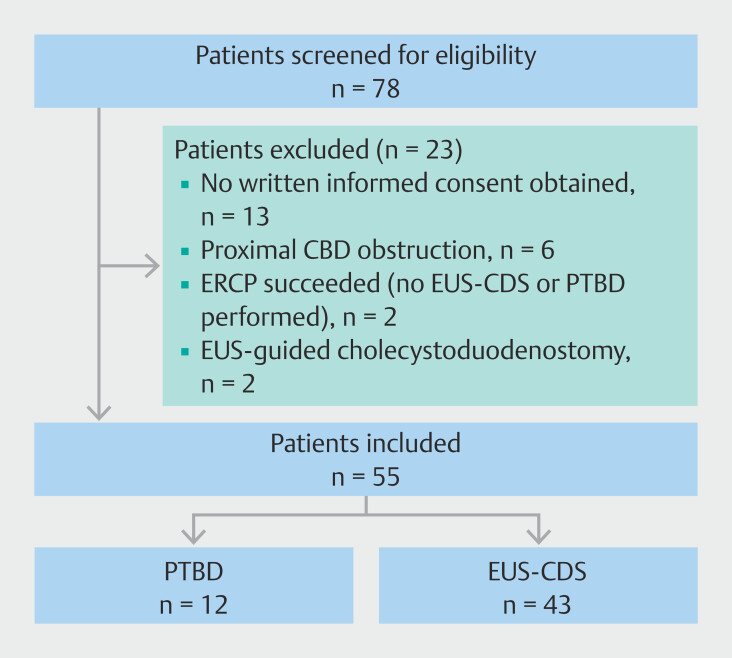
Flowchart of patient inclusion. CBD, common bile duct; EUS-CDS, endoscopic ultrasound-guided choledochoduodenostomy; PTBD, percutaneous transhepatic biliary drainage.

We screened 78 patients for eligibility between July 2022 and December 2023. Of these, 13 patients did not provide written informed consent, mainly because of terminal illness. Two patients were scheduled for same-session ERCP with, if necessary, an EUS-CDS, but the ERCP procedure was successful. Six patients had a proximal CBD obstruction and EUS-guided cholecystoduodenostomy was performed in two other patients; therefore, these eight patients were not included in this study. None of the patients screened for eligibility underwent EUS-guided rendezvous or antegrade stenting after their failed ERCP. Finally, 55 patients were included: 12 underwent PTBD and 43 patients EUS-CDS ( Fig. 2 ). The number of screened and included patients in each hospital is shown in **Table 1s** , see online-only Supplementary material.

Among the 12 patients who underwent PTBD, PTBD was chosen as the primary drainage modality after the failure of ERCP, and there was no intention to perform EUS-CDS. There were 5/12 patients who were not eligible for EUS-CDS owing to ascites, collateral vessels, or a CBD with too small a diameter on previous imaging. An additional five patients were unable to undergo EUS-CDS owing to nonavailability of an endoscopist who was proficient in EUS-CDS. Additionally, PTBD was performed in two patients with a stenosis of the upper digestive tract, which had already been observed during their failed ERCP ( **Fig. 1s** ).

### Baseline characteristics

The baseline characteristics of the two groups were quite similar ( Table 1 ), except for a slightly higher percentage of men in the PTBD group (75.0% vs. 46.5%). The patients’ median age was 71 years (interquartile range [IQR] 64–76 years) and their median body mass index (BMI) was 24.8 kg/m ^2^ (IQR 23.3–28.5 kg/m ^2^ ). Obstruction of the CBD was predominantly caused by pancreatic adenocarcinoma (65.5%), followed by distal cholangiocarcinoma (10.9%) and metastatic tumors (10.9%). Fourteen patients (25.5%) had a primary resectable tumor (two patients in the PTBD group [16.7%] and 12 in the EUS-CDS group [27.9%]). Both patients in the PTBD group and 13 patients in the EUS-CDS group underwent surgery: while one patient with a primary resectable tumor in the EUS-CDS group did not undergo surgery, two others with a borderline resectable tumor did undergo surgery after treatment with neoadjuvant chemotherapy.

**Table:** **Table 1** Baseline characteristics of the patients with failed endoscopic retrograde cholangiopancreatography (ERCP) for drainage of distal malignant biliary obstruction who were included in the study.

	Total (n = 55)	PTBD (n = 12)	EUS-CDS (n = 43)
Age, median (IQR), years	71 (64–76)	72 (63–77)	71 (64–76)
Sex, male, n (%)	29 (52.7)	9 (75.0)	20 (46.5)
BMI, median (IQR), kg/m ^2 1^	24.8 (23.3–28.5)	26.6 (23.3–31.7)	24.5 (23.0–28.1)
Type of cancer, n (%)			
Pancreatic adenocarcinoma	36 (65.5)	8 (66.7)	28 (65.1)
Distal cholangiocarcinoma	6 (10.9)	0 (0.0)	6 (14.0)
Ampullary cancer	3 (5.5)	1 (8.3)	2 (4.7)
Metastasis of other primary tumor	6 (10.9)	1 (8.3)	5 (11.6)
Other	4 (7.3)	2 (16.7)	2 (4.7)
ASA class, n (%)			
I	2 (3.6)	0 (0.0)	2 (4.7)
II	26 (47.3)	7 (58.3)	19 (44.2)
III	27 (49.1)	5 (41.7)	22 (51.2)
Smoking, n (%)			
No	24 (43.6)	7 (58.3)	17 (39.5)
Yes, former	24 (43.6)	5 (41.7)	19 (44.2)
Yes, current	7 (12.7)	0 (0.0)	7 (16.3)
Anticoagulant use			
None	39 (70.9)	8 (66.7)	31 (72.1)
DOAC	6 (10.9)	1 (8.3)	5 (11.6)
Antiplatelet	4 (7.3)	0 (0.0)	4 (9.3)
LMWH	4 (7.3)	3 (25.0)	1 (2.3)
Vitamin-K antagonists	2 (3.6)	0 (0.0)	2 (4.7)
Alcohol intake, median (IQR), units/week	5 (1–14)	3 (1–7)	7 (2–14)
Cholangitis at time of procedure, n (%)	3 (5.5)	1 (8.3)	2 (4.7)
Reason failed ERCP, n (%)			
Could not reach papilla	12 (21.8)	4 (33.3)	8 (18.6)
Could not cannulate CBD	35 (63.6)	7 (58.3)	28 (65.1)
CBD cannulation achieved but impossible to insert guidewire	7 (12.7)	0 (0.0)	7 (16.3)
Impossible to place stent	1 (1.8)	1 (8.3)	0 (0.0)
Intended treatment if ERCP successful, n (%)			
Surgery	14 (25.5)	2 (16.7)	12 (27.9)
Palliative chemotherapy	14 (25.5)	3 (25.0)	11 (25.6)
Neoadjuvant chemotherapy	8 (14.5)	0 (0.0)	8 (18.6)
Best supportive care	19 (34.5)	7 (58.3)	12 (27.9)
ASA, American Society of Anaesthesiologists; BMI, body mass index; CBD, common bile duct; DOA, direct oral anticoagulants; IQR, interquartile range; LMWH, low molecular weight heparin. ^1^ Data not avialable for three patients.			

At initial diagnosis, 33 patients (60.0%) were in a palliative condition (10 in the PTBD group [83.3%] and 23 in the EUS-CDS group [53.5%]). Prior to ERCP, most patients had opted for best supportive care (19/55; 34.5%), with a higher preference in the PTBD group (7/12; 58.3%) than in the EUS-CDS group (12/43; 27.9%).

The main reason for ERCP failure was an inability to achieve CBD cannulation (63.6%). Furthermore, in 21.8% of patients, the major papilla could not be reached owing to a stenosis in the upper digestive tract, including one patient who had esophageal obstruction, one with type I duodenal obstruction, and 10 with type II obstruction. In the latter 10 patients, the residual duodenal wall was adequate for performance of an EUS-CDS.

### PTBD and EUS-CDS procedures

A total of 12 PTBD procedures were performed, with a technical success rate of 100%, whereas the technical success rate of EUS-CDS was 97.7% (42/43 procedures). The median interval to performance of the alternative procedure after the failed ERCP was 4 days (IQR 1–7 days) in the PTBD group and 1 day (IQR 0–4 days) in the EUS-CDS group ( *P* = 0.08). A 10-Fr drain was predominantly used for PTBD (n = 9; 81.8%), with the drains mainly placed through the right liver lobe (58.3%). None of the patients underwent placement of a SEMS during the initial PTBD session. For two patients in the PTBD group and two in the EUS-CDS group, two attempts were required during one session to access the bile duct.

Except for two EUS-CDS procedures, freehand insertion was the most frequently used technique to place a stent between the duodenum and CBD. The most commonly placed stent type was an 8 × 8-mm LAMS (n = 32; 74.4%). In 26/43 EUS-CDS procedures (60.5%), a double-pigtail stent was inserted through the LAMS.

### Outcomes and AEs

During the procedure, one LAMS was misdeployed into the dilated CBD. In both groups, one bleed occurred, with both being self-limiting. The median duration of hospital stay was significantly shorter after an EUS-CDS procedure compared with a PTBD procedure (1 day [IQR 1–2 days] vs. 4 days [IQR 2–6 days]; *P* = 0.001) ( Table 2 ).

**Table:** **Table 2** Comparison of the technical characteristics for the endoscopic ultrasound-guided choledochoduodenostomy (EUS-CDS) and percutaneous transhepatic biliary drainage (PTBD) procedures.

	PTBD (n = 12)	EUS-CDS (n = 43)	*P* value
Technical success, n (%)	12 (100%)	42 (97.7%)	>0.99
Interval between ERCP and procedure, median (IQR), days	4 (1–7)	1 (0–4)	0.08
Same-session as ERCP procedure, n (%)	0 (0.0)	21 (48.8)	0.002
Attempts to access to bile duct, n (%)			0.20
1	10 (83.3)	41 (95.3)	
2	2 (16.7)	2 (4.7)	
Location of puncture, n (%)			–
Right liver lobe	7 (58.3)	N/A	
Left liver lobe	5 (41.7)		
Antibiotics administered prior to PTBD, n (%) ^1^	8 (72.7)	N/A	–
Size drain, n (%) ^1^			–
7 Fr	1 (9.1)	N/A	
8 Fr	1 (9.1)		
10 Fr	9 (81.8)		
Technique, n (%)			–
Needle and wire	N/A	2 (4.7)	
Freehand insertion		41 (95.3)	
Size of LAMS used, n (%), mm			–
8 × 8	N/A	32 (74.4)	
6 × 8		9 (20.9)	
10 × 10		1 (2.3)	
10 × 20		1 (2.3)	
Pigtail stent inserted through LAMS, n (%)	N/A	26 (60.5)	–
Hospital admission post-procedure, median (IQR), days	4 (2–6)	1 (1–2)	0.001
Periprocedural AEs, n (%) ^2^	1 (8.3)	2 (4.7)	0.53
AE rate <72 hours post-procedure, n (%)	4 (33.3)	9 (20.9)	0.45
Grade I AEs <72 hours post-procedure, n (%)			0.30
Bile leakage	1 (8.3)		
Electrolyte disturbance		1 (2.3)	
Nausea and vomiting		2 (4.7)	
Severe post-procedural pain	1 (8.3)		
Grade II AEs <72h post-procedure, n (%)			0.64
Acute pancreatitis	1 (8.3)		
Bleeding		1 (2.3)	
Cholangitis	1 (8.3)		
Fever		1 (2.3)	
Electrolyte disturbance		1 (2.3)	
Severe post-procedural pain		1 (2.3)	
Hypotension		1 (2.3)	
Grade V AEs <72h post-procedure, n (%)			–
Acute kidney injury	0 (0.0)	1 (2.3)	
AE, adverse event; ERCP, endoscopic retrograde cholangiopancreatography; IQR, interquartile range; LAMS, lumen-apposing metal stent. ^1^ Data not available for one patient. ^2^ Periprocedural AEs were: PTBD group, bleeding (n = 1, grade I); EUS-CDS group, bleeding (n = 1, grade II) and stent misdeployment into CBD (n = 1, grade II).			

Within 72 hours post-procedure, one patient died in the EUS-CDS group owing to bile cast nephropathy. One or more AEs occurred in 4/12 patients (33.3%) in the PTBD group and 9/43 patients (20.9%) in the EUS-CDS group. In the PTBD group, 2/4 AEs were grade II AEs, compared with 5/9 AEs in the EUS-CDS group ( *P* = 0.64). The two grade II AEs in the PTBD group were acute pancreatitis and cholangitis, while administration of antibiotics for fever of unknown origin and administration of blood products resulted in two of the grade II AEs in the EUS-CDS group. Prolonged hospital admission owing to hypotension, severe post-procedural pain, and electrolyte disturbances resulted in the remaining three grade II AEs. Other AEs were all grade I AEs.

At the end of 30 days post-procedure, a reduction of >50% in the total bilirubin level was achieved in 10/12 patients (83.3%) in the PTBD group compared with 23/31 patients (74.2%) in the EUS-CDS group ( *P* = 0.70).

None of the adequately placed LAMSs migrated within 90 days. A total of eight LAMSs (19.0%) became obstructed within 90 days by food or medication impaction, resulting in five therapeutic duodenoscopies for LAMS inspection and, if needed, debridement. Additionally, in one patient a SEMS was placed via the transpapillary route by ERCP, and in another patient a plastic stent was similarly placed, followed by LAMS removal. Furthermore, one patient underwent earlier pancreaticoduodenectomy. Among the obstructed LAMSs, three had a double-pigtail plastic stent in place, while five did not. The obstruction rate was 11.5% (3/26) for LAMSs with a double-pigtail plastic stent compared with 31.3% (5/16) for those without. In the PTBD group, seven drains required exchange or removal, and three SEMSs were placed via the percutaneous route. In the first 30 days, 29 patients could not undergo an oncological treatment. In four patients, the postponement of the treatment was caused by AEs due to the PTBD or EUS-CDS, and eight patients had already died.

In the 90 days after the procedures, 11 patients in the PTBD group (91.7%) had one or more AEs compared with 19 patients in the EUS-CDS group (44.2%; *P* = 0.004). Cholangitis was the most common infectious complication, resulting in four grade II, eight grade III, and two grade V AEs. The incidence of grade III cholangitis was significantly higher in the PTBD group (25.0% vs. 11.6%; *P* = 0.04). The most frequently encountered noninfectious AEs in the PTBD group were bile leakage (33.3%) and hemorrhage (16.7%). In the EUS-CDS group, reflux, and constipation and/or diarrhea were the two leading noninfectious complications (16.3% and 11.6%, respectively) ( Table 3 ).

**Table:** **Table 3** Comparison of clinical outcomes and adverse events (AEs) in the endoscopic ultrasound-guided choledochoduodenostomy (EUS-CDS) and percutaneous transhepatic biliary drainage (PTBD) groups.

	Total (n = 55)	PTBD (n = 12)	EUS-CDS (n = 43)	*P* value
Clinical success, n (%)	33/43 (76.7)	10/12 (83.3)	23/31 (74.2)	0.70
Mortality <90 days, n (%)	17 (30.9)	8 (66.7)	9 (20.9)	0.005
Stent occlusion, n (%)				–
<30 days	N/A	N/A	3/42 (7.1)	
30–90 days			5/29 (17.2)	
Stent migration after adequate placement of LAMS, n (%)				–
<30 days	N/A	N/A	0/42 (0)	
30–90 days			0/29 (0)	
Reinterventions before 90 days, n (%)				<0.001
Therapeutic duodenoscopy	N/A		5 (11.6)	
Transpapillary stent placement after LAMS removal			2 (4.7)	
Upfront surgery			1 (2.3)	
Drain removal/exchange		7 (58.3)		
Stent placement after PTBD by interventional radiologist		3 (25.0)		
Scheduled reinterventions	N/A	8 (66.7%)	0 (0.0)	<0.001
Length of readmisions before 90 days, median (IQR), days	N/A	3 (0–13)	0 (0–5)	0.07
Grade I AEs <90 days, n (%)				0.07
Abscess	1 (1.8)	1 (8.3)		
Bile leakage	3 (5.5)	3 (25.0)		
Constipation and/or diarrhea	4 (7.3)	1 (8.3)	3 (7.0)	
Electrolyte disturbance	1 (1.8)		1 (2.3)	
Hypotension	1 (1.8)		1 (2.3)	
Reflux	4 (7.3)		4 (9.3)	
Severe postprocedural pain	1 (1.8)	1 (8.3)		
Grade II AEs <90 days, n (%)				0.45
Acute pancreatitis	1 (1.8)	1 (8.3)		
Bleeding	1 (1.8)		1 (2.3)	
Cholangitis	4 (7.3)	3 (25.0)	1 (2.3)	
Cholecystitis	1 (1.8)		1 (2.3)	
Constipation and/or diarrhea	1 (1.8)		1 (2.3)	
Fever	1 (1.8)		1 (2.3)	
Electrolyte disturbances	1 (1.8)		1 (2.3)	
Severe post-procedural pain	1 (1.8)		1 (2.3)	
Hypotension	1 (1.8)		1 (2.3)	
Peripancreatic inflammation	1 (1.8)		1 (2.3)	
Grade III AEs <90 days, n (%)				0.04
Abscess	1 (1.8)		1 (2.3)	
Bile leakage	1 (1.8)	1 (8.3)		
Biloma	1 (1.8)	1 (8.3)		
Bleeding	2 (3.6)	2 (16.7)		
Cholangitis	8 (14.5)	3 (25.0)	5 (11.6)	
Cholecystitis	1 (1.8)		1 (2.3)	
Constipation and/or diarrhea	1 (1.8)		1 (2.3)	
Hematemesis	2 (3.6)	1 (8.3)	1 (2.3)	
Reflux	3 (5.5)		3 (7.0)	
Severe postprocedural pain	1 (1.8)		1 (2.3)	
Grade IV AEs <90 days, n (%)				–
Cerebrovascular accident	1 (1.8)		1 (2.3)	
Grade V AEs <90 days, n (%)				>0.99
Acute kidney injury	1 (1.8)		1 (2.3)	
Cholangiosepsis	2 (3.6)	1 (8.3)	1 (2.3)	
Overall AE rate <90 days, n (%)	30 (54.5)	11 (91.7)	19 (44.2)	0.004
Quality of life after 30 days, mean (SD) ^1^				
SF36 – Physical component scale	N/A	39.9 (12.1)	39.2 (6.9)	0.90
SF36 – Mental component scale		45.8 (8.6)	41.6 (7.5)	0.42
EQ-5D		0.73 (0.20)	0.65 (0.18)	0.36
Quality of life after 90 days, mean (SD) ^2^				
SF36 – Physical component scale	N/A	37.1 (8.4)	40.6 (9.6)	0.47
SF36 – Mental component scale		52.8 (7.4)	43.7 (6.3)	0.04
EQ-5D		0.86 (0.05)	0.74 (0.14)	0.15
EQ-5D, EuroQol-5 dimensions; ERCP, endoscopic retrograde cholangiopancreatography; LAMS, lumen-apposing metal stent; SF-36, Short Form Health Survey-36. ^1^ Data from 5 and 21 participants in the PTBD and EUS-CDS groups, respectively. ^2^ Data from 3 and 20 participants in the PTBD and EUS-CDS groups, respectively.				

**Fig. 3:**
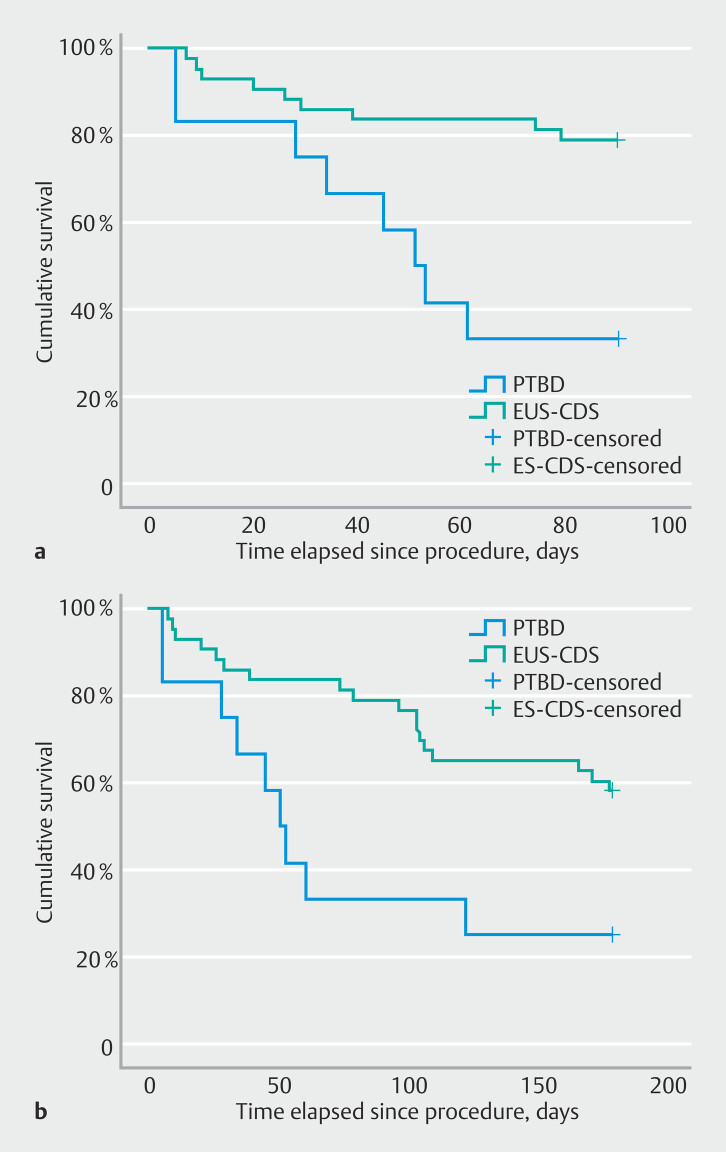
Kaplan–Meier curves of survival after percutaneous transhepatic biliary drainage (PTBD) or endoscopic ultrasound-guided choledochoduodenostomy (EUS-CDS) at: **a** 90 days (log rank [Mantel–Cox] test, *P* = 0.001); **b** 180 days ( *P* = 0.008).

Mortality rates at both 90 and 180 days post-procedure were significantly higher in the PTBD group compared with the EUS-CDS group ( *P* = 0.001 and *P* = 0.008, respectively) ( Fig. 3 ). After 90 days, 66.7% of the patients in the PTBD were deceased compared with 20.9% in the EUS-CDS group.

### Quality of life

QoL data at 30 and 90 days post-procedure were available for only five and three patients in the PTBD group, and 21 and 20 patients in the EUS-CDS group, respectively. SF-36 scores at both timepoints were below average with a slight improvement in mental QoL observed between 30 and 90 days (increasing from 45.8 [SD 8.6] to 52.8 [SD 7.4] in the PTBD group and from 41.6 [SD 7.5] to 43.7 [SD 6.3] in the EUS-CDS group). Similarly, the EQ-5D scores showed an improvement in both groups between 30 and 90 days post-procedure, with mean differences of 0.13 and 0.09, respectively ( *P* = 0.15).

## Discussion

In this registry, we monitored the clinical outcomes of 55 patients who underwent PTBD or EUS-CDS after a failed ERCP over a period of 180 days, or until death or pancreaticoduodenectomy. Our study showed lower AE, mortality, and reintervention rates in the EUS-CDS group compared with the PTBD group. Both groups demonstrated comparable high technical and clinical success rates. These results are in alignment with data from a previously conducted randomized controlled trial, with technical success of around 95% 24 . Furthermore, pooled data in a meta-analysis supported this finding, without significant difference between the two modalities 17 .

Various studies have reported 30-day mortality rates associated with PTBD, ranging between 16.8% and 23.1% 8 9 12 . Mortality with EUS-CDS has been mainly expressed as median survival in several studies; however, a recently published prospective cohort study demonstrated a 30-day mortality of 4.5% 16 . To further assess the long-term effectiveness of EUS-CDS compared with PTBD, we extended our observation period to 90 days. After 90 days, the mortality rates were 66.7% in the PTBD group and 20.9% in the EUS-CDS group. The PTBD group probably consisted of patients in poorer clinical condition, who also more frequently opted for best supportive care, compared with the patients in the EUS-CDS group. Ascites, which is often a manifestation of peritoneal metastasis and is correlated with rapid deterioration in a patient’s condition 25 , can complicate performing EUS-CDS 13 , which possibly led to prompter PTBD, with or without same-session ascites drainage.

None of the stents migrated, but stent occlusion occurred in eight LAMSs (19.0%), which is lower than the rates reported in the literature (31.8%–55%) 16 26 . In both these studies, predominantly 6 × 8-mm LAMSs were inserted, whereas in our study mainly 8 × 8-mm LAMSs were inserted. The 8 × 8-mm LAMS is associated with fewer AEs compared with the 6 × 8-mm LAMS 27 . Furthermore, both studies reported LAMS placement mainly without the insertion of additional transluminal double-pigtail plastic stents 16 26 . In our study, 5/8 obstructed LAMSs did not have a transluminal double-pigtail plastic stent; a LAMS with a transluminal double-pigtail plastic stent has been reported to be superior to placement of a LAMS alone for prevention of stent obstruction 28 .

In each group, cholangitis was diagnosed seven times (i.e. 58.3% in the PTBD group and 16.3% in the EUS-CDS group). As shown by others, the risk of acute pancreatitis after EUS-CDS is negligible 16 29 , while we observed one case of acute pancreatitis in the PTBD group. As the risk of obstructing the cystic duct by LAMS placement is increased 30 , we also observed two cases (4.7%) of acute cholecystitis, a rate lower than the 10% previously described by Fritzsche et al. 31 ; however, in this study a SEMS was placed through the LAMS in 83.3% of cases, which might have occluded the cystic duct 31 .

The aim of our study was also to gain insight into which of the drainage modalities was preferred by the attending physician when both modalities were available. In our study, the attending physician preferred EUS-CDS over PTBD, unless it was technically impossible to create a choledochoduodenostomy or an experienced interventional endoscopist was not available. Most of the participating endoscopists who performed the ERCP were also able to perform the EUS-CDS, leading to same-session failed ERCP followed by EUS-CDS. This is also reflected in the shorter hospital stay in the EUS-CDS group compared with the PTBD group. Another advantage of same-session EUS and ERCP is the ability to perform fine needle biopsy (FNB) to obtain histology for the diagnosis of pancreatic cancer. In our cohort, it was not possible to perform PTBD on the same day as the unsuccessful ERCP, with PTBD being performed a median of 4 days later. Additionally, the median hospital stay after PTBD was 3 days longer than it was following EUS-CDS.

One of the strengths of our cohort is the inclusion of patients from 15 different hospitals across the Netherlands. Most of these centers were not academic expert centers. By including patients from both academic and community hospitals, we enhanced the generalizability of the outcomes, representing real-world data. In addition, we did not analyze only the clinical outcomes and AEs associated with both EUS-CDS and PTBD, but also investigated the rationale behind the selection of PTBD over EUS-CDS. The preference of gastroenterologists for EUS-CDS over PTBD is consistent with patient preferences, as described in an international multicenter survey conducted by Nam et al. 32 .

We observed an improvement in QoL between 30 and 90 days post-procedure, which may be attributed to patient’s psychological disease acceptance 33 ; however, the small sample size in the PTBD group, with only three patients completing the 90-day QoL questionnaires, limits the robustness of our conclusions about QoL. The small sample size also limited our ability to perform additional analyses, such as multivariate regression analysis, to identify risk factors for developing AEs.

Another limitation of the study was that the gastroenterologist who performed the ERCP was often also trained to perform an EUS-CDS, allowing an immediate decision regarding suitability of patients for EUS-CDS. This could have introduced selection bias, as only patients considered unsuitable for EUS-CDS were subsequently referred for PTBD. This is reflected in differences in the baseline characteristics of the groups, as patients who underwent PTBD had a higher incidence of unresectable tumors, had opted more frequently for best supportive care, and were therefore possibly in a worse clinical condition. Additionally, given that local investigators were responsible for patient screening and inclusion, some further selection bias may also have been introduced.

While our study focused on comparing EUS-CDS and PTBD, it is worth mentioning that other EUS-BD techniques, such as EUS-guided rendezvous, EUS-guided hepaticogastrostomy and antegrade stenting, are also possible alternatives 11 34 . Especially if there is gastric outlet obstruction, antegrade stent placement or EUS-guided hepaticogastrostomy are appropriate options, while EUS-CDS is often technically not possible 35 . These techniques are widely used in expert centers and provide additional alternatives for achieving biliary drainage after failed ERCP instead of PTBD 13 . Nevertheless, these alternative EUS-BD techniques were not performed in our study cohort, as the focus was on EUS-CDS as the primary alternative endoscopic drainage method.

In conclusion, when both PTBD and EUS-CDS are available and technically feasible, EUS-CDS is favorable as it seems to be associated with fewer AEs, lower mortality, fewer reinterventions, and a shorter hospital admission time compared with PTBD after a failed ERCP in patients with a distal MBO. Training therapeutic endoscopists in both ERCP and EUS-BD is therefore a worthwhile strategy to ensure optimal outcomes for biliary drainage.

## Supplementary Material

Supplementary Material Zusatzmaterial
