# Supplementary material for: Endoscopic ultrasound-guided choledochoduodenostomy results in fewer complications than percutaneous drainage following failed ERCP in malignant distal biliary obstruction
Source: Endoscopy. 2025 May 21;57(9):1004–15. doi: 10.1055/a-2580-1316 (PMC12417859; doi:10.1055/a-2580-1316)
Supplement: Supplementary file 1 — Supplementary Material [file 10-1055-a-2580-1316_25990885.pdf]

SUPPLEMENTARY MATERIAL

EUS-guided choledochoduodenostomy is associated with fewer adverse events compared to PTBD after failed ERCP in patients with distal malignant biliary obstruction.

Mike J.P. de Jong, Foke van Delft, Erwin-Jan M. van Geenen, Auke Bogte, Robert C. Verdonk, Niels G. Venneman, Jan Maarten Vrolijk, Jan-Willem A. Straathof, Rogier P. Voermans, Rina A. Bijlsma, Sjoerd D. Kuiken, Rutger Quispel, Muhammed Hadithi, Kirill Basiliya, Frank P. Vleggaar, Tanya M. Bisseling, Thomas R. de Wijkerslooth, Marco J. Bruno, Roy L.J. van Wanrooij and Peter D. Siersema

Table 1s Patients screened and included at each hospital

Hospital	Type of hospital	Patients screened	Patients included
Amsterdam University Medical Center	Academic hospital	15	10
St. Antonius Hospital	Community hospital	11	5
Antoni van Leeuwenhoek Hospital	Community hospital	1	1
Erasmus University Medical Center	Academic hospital	2	0
Isala Hospital	Community hospital	0	0
Maasstad Hospital	Community hospital	4	4
Martini Hospital	Community hospital	4	4
Maxima Medical Centre	Community hospital	2	1
Medisch Spectrum Twente	Community hospital	6	5
Onze Lieve Vrouwe Gasthuis	Community hospital	1	1
Radboud university medical center	Academic hospital	17	15
Reinier de Graaf Hospital	Community hospital	2	1
Rijnstate Hospital	Community hospital	3	3
University Medical Centre Utrecht	Academic hospital	10	5
**TOTAL**		**78**	**55**

Figure 1s Indication PTBD instead of EUS-CDS

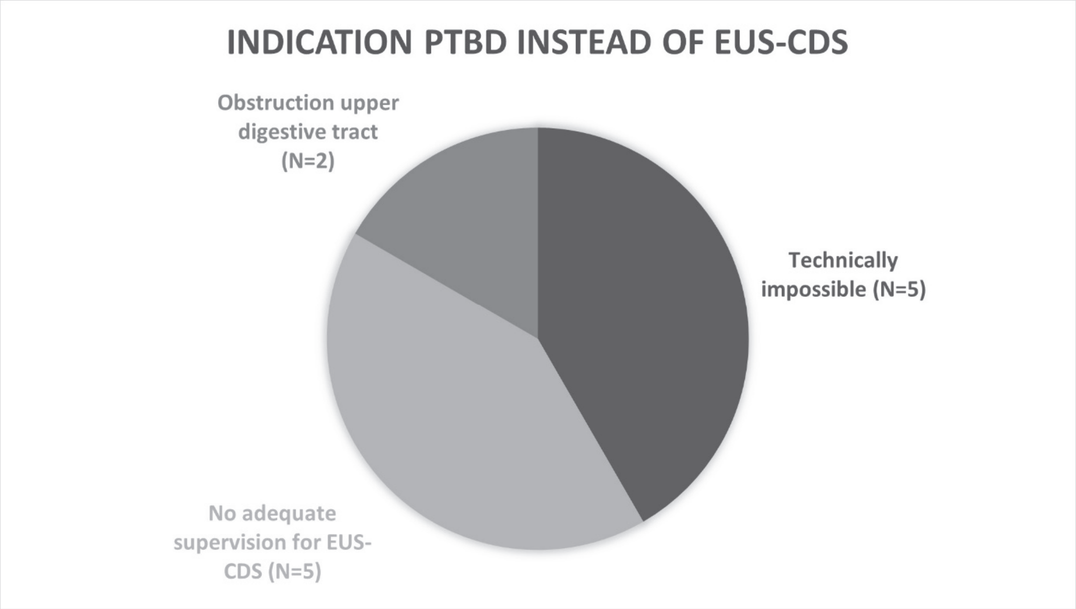

PTBD = percutaneous transhepatic biliary drainage. EUS-CDS = endoscopic ultrasound guided choledochoduodenostomy.
